# External Quality Assessment Programme for Early Infant Diagnosis of HIV-1, Mozambique, 2011–2014

**DOI:** 10.4102/ajlm.v7i1.664

**Published:** 2018-10-11

**Authors:** Nalia Ismael, Orvalho Augusto, Adolfo Vubil, Sofia O. Viegas, Fernanda Miambo, Patrina Chongo, Nédio Mabunda

**Affiliations:** 1 Instituto Nacional de Saúde, Maputo, Mozambique; 2 Faculty of Medicine, Eduardo Mondlane University, Maputo, Mozambique; 3 Instituto Superior de Ciência e tecnologia de Moçambiq, Maputo, Mozambique

## Abstract

This study evaluated a National External Quality Scheme Program for early infant diagnosis of HIV. Fourteen laboratory technicians participated and nine testing panel cycles were sent between 2011 and 2014. The response rate was 100% for the first eight panels, and the number of technicians with a test score of 100% increased during the first three panels. Based on the evaluations of the technicians, the quality of testing for early infant diagnosis of HIV improved over time in the laboratories.

## Introduction

Early infant diagnosis (EID) of HIV infection assures early access to antiretroviral therapy for infected children, which significantly improves survival rates and provides substantial benefits.^1,2^ The antibody-specific methods, such as enzyme immunological assays used to diagnose HIV infection in adults, are not recommended for infant diagnosis, due to the passive transfer of antibodies by the mother to the baby in the uterus until the age of 18 months. Nucleic acid tests such as HIV RNA and DNA polymerase chain reaction (PCR) assays are recommended to diagnose HIV in newborns.^3,4^

In settings that lack adequate infrastructure and cold-chain transportation systems to process whole blood, dried blood spot (DBS) samples offers many advantages. Dried blood spot testing has simplified HIV screening in newborns, because samples are easy to collect often via heel prick or finger stick and no cold chain transportation system is required as DBS samples are stable at room temperature.^5,6,7,8,9,10,11^

Participation in an external quality assurance (EQA) programme has been shown to detect possible errors during testing, trace possible corrective actions and improve the quality of testing, and is thus an important tool for laboratory quality assurance.^12,13,14,15,16,17,18^ Available molecular tests for newborns have high sensitivity and specificity when performed correctly but when the procedures recommended by the manufacturers are not correctly performed it can result in incorrect results, which has serious consequences. Incorrect results may lead to wrong clinical follow-up and a delay in treatment initiation.^19,20^ To ensure the accuracy and reliability of laboratory test results, participation in an EQA programme is crucial.^21^

Since 2006, Mozambique has been using PCR-based methods with DBS samples to diagnose HIV in exposed newborns. In 2011, the *Instituto Nacional de Saúde* of Mozambique, through the National EQA Program, introduced a voluntary EQA scheme designated for each laboratory technician performing PCR-based EID on DBS samples. This report describes the results of four years of evaluation of the National EQA Program scheme in Mozambique.

## Methods

### Ethical considerations

The ethics committee of the *Instituto Nacional de Saúde*, Maputo, Mozambique approved the study (study number: 162/CIBS-INS/2017).

### Implementation of external quality assurance HIV-1 DNA programme

Between 2011 and 2014, all laboratory technicians performing EID for HIV using DBS were informed about the availability of proficiency testing panels provided by the National EQA Program of Mozambique and were encouraged to enroll and participate. The panels were provided free of charge and sent to each technician for testing within four weeks of receipt. To maintain confidentiality, a unique code was assigned to each technician.

### Panel description and preparation

The proficiency testing panels consisted of 20 blinded DBS samples a mixture of negative and positive samples. Negative DBS samples were prepared from a negative blood bag collected by the Blood Bank at *Hospital Central de Maputo*. Positive DBS samples were prepared from an HIV-positive individual’s blood used for CD4 testing at the Cellular Immunology Laboratory in the same hospital. The positive and negative samples were retested using the Amplicor HIV-1 DNA PCR test, version 1.5 (Roche Molecular Systems, Branchburg, New Jersey, United States), according the manufacturer’s instructions.

### Proficiency panel description and sample organisation

Four series were prepared, and each series consisted of 20 blinded DBS samples in a different sample arrangement. Negative and positive specimens were prepared separately, dried overnight, then encoded with a sample and serial number. Each series contained 20 DBS specimens packed into a sealable plastic storage bag stored at 2°C – 8°C until shipment. All four series were sent to each laboratory, and each laboratory technician had to test one of the series sent.

### Panel validation

To validate the panel, each series was selected randomly and all 20 DBS samples within the series were retested using the Amplicor HIV-1 DNA PCR test, version 1.5 (Roche, Germany) according to the manufacturer´s instructions, by two different technicians. The results were crosschecked and both results had to match. Following validation, the proficiency panels were sent and made available to all the laboratories across the country performing EID diagnosis.

### Sample shipment, analysis, results and reporting

A carrier company shipped the proficiency panels at room temperature three times during 2011 and twice between 2012 and 2014. The technicians had to report the results within 30 days after panel reception. All technicians that did not report their results within 30 days after receiving the panels were considered to be non-responders.

Test scores were reported in percentages according to the number of correct results out of the 20 tests per cycle. Technicians were evaluated according to the concordance of their results with expected results established previously. After each panel, each technician was sent a report containing (1) the test scores (percentage of concordant results), (2) reported results, as well as expected results and possible causes in cases of discordant results. For technicians with results below 100%, corrective actions were conducted, including technical support through laboratory visits, emails or telephone calls.

### Statistical analysis

Frequencies and proportions were used to describe the data. Trends between 2011 and 2014 were studied by means of graphs or tables. To study the association between the proportion of technicians scoring 100% on a panel and concordance among technicians, we use a beta-binomial regression with a logit link. We chose this regression to keep the fitted concordance within the 0 to 1 range and to respect the uncaptured geographic heterogeneity (over dispersion) of the data. Based on the Akaike information criterion and Bayesian information criterion, the proportion of technicians scoring 100% was included as linear and quadratic transformed. The quadratic terms were included to detect U-shaped relationships in the data. Stata 14 (StataCorp. 2015. Stata: Release 14. Statistical Software; StataCorp LP, College Station, Texas, United States) was used to perform all analysis.

## Results

From 2011 to 2014, nine proficiency panels were sent to each technician. The overall number of technicians that participated increased from 10 in 2011 to 12 in 2014 (Table 1). During the course of the study, there were some withdrawals and replacements for all laboratories with the exception of Laboratory A, where the same technicians were evaluated during the whole course of the study (Table 1). For all panels, results were sent within 30 days of receipt of the panels (78.5% – 100%; average: 97.6%), with the exception of one test panel (2014-B) because three technicians did not respond.

**TABLE 1:** Overall number of technicians that participated in the National External Quality Assessment Program for polymerase chain reaction of HIV DNA across the nine panels, Mozambique, 2011–2014.

Panel number	Panel name	Number of technicians that participated	Response rate %
1	2011-A	10	100
2	2011-B	11	100
3	2011-C	12	100
4	2012-A	13	100
5	2012-B	13	100
6	2013-A	13	100
7	2013-B	13	100
8	2014-A	14	100
9	2014-B	14	78.5
-	Average	12.6	97.6

%, percentage.

For Laboratory D, 18 errors were observed, and 100% of the technicians had at least one error during the nine panels (Table 2). Laboratory B had the least errors with 2, followed by Laboratory C with 10. At least one error occurred for 16.6% of technicians in Laboratory B and 75% of technicians in Laboratory C. Laboratory A had the second-highest number of errors and 20% of technicians had at least one error.

**TABLE 2:** Error characterisation of technicians per laboratory during the nine cycles.

Laboratory	Error types					
	False positives	False negatives	Total invalids/indetermined	Total errors	Number of technicians enrolled by 2014	Percentage of technicians with at least one error during the nine cycles
Laboratory A	4	6	2	12	4	20
Laboratory B	0	2	0	2	3	16.60
Laboratory C	0	8	2	10	4	75
Laboratory D	11	4	3	18	3	100

**FIGURE 1:**
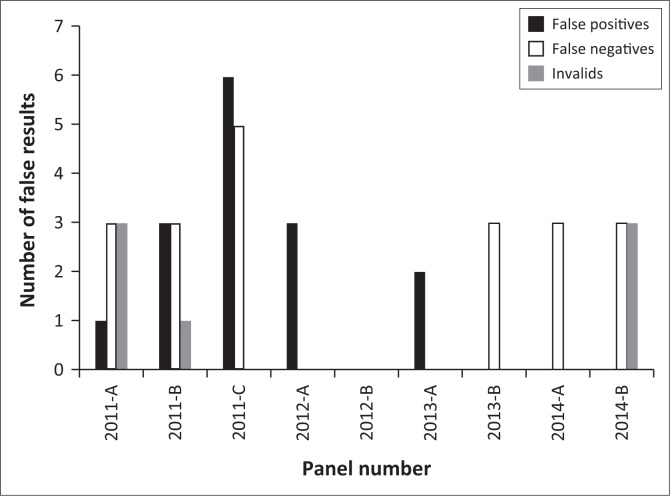
Percentage of discordant results among all participating technicians. Discordant results included false positives, false negatives and invalids.

**FIGURE 2:**
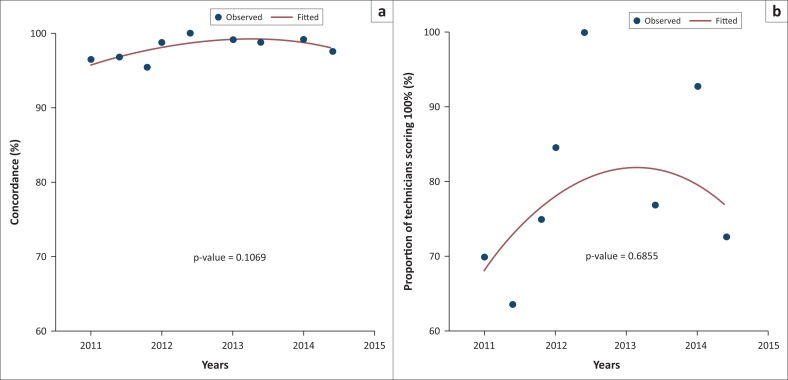
Percentage of technicians scoring 100% versus their concordance during each test panel from 2011–2014. The line is a fit of a beta-binomial regression with linear and quadratic terms of technicians scoring 100%.

The number of false positives increased during the first three panels, while no false positives were observed during the last three panels (2013-B, 2014-A and 2014-B) (Figure 1). On the other hand, the number of false negatives was constant for panels 2011-A, 2011-B, 2011-C, 2013-B, 2014-A and 2014-B. Invalid results were observed during the second panels and re-emerged during the last panel (2014-B). During the first three panels, the proportion of technicians scoring 100% increased, but decreased by 2% during the last two panels (Figure 2).

## Discussion

During the expansion of EID testing and treatment programmes in Mozambique, it was necessary to optimise the quality testing for HIV DNA PCR. We report the successful implementation of the HIV-1 EID EQA Program in Mozambique during the period of 2011 to 2014. Our results show that the quality testing slightly improved over time after the EID EQA program was implemented. If progress is to be maintained, EID EQA program should be sustained.

The number of technicians participating increased during the first eight panels of the programme, with a response rate of 100%, suggesting a positive response and growing interest in the programme. Initially, training on EID for HIV conducted by the laboratory that provided the proficiency testing panels and the clear explanation of the objectives and benefits of the programme to its participants may have accounted for the increased participation. For each panel, technicians with discordant results had direct site supervision and indirect technical support (via email or telephone). The decrease in the response rate during the last panel was most likely related to annual leave during the period that the panel was sent. As the number of samples tested in the country for EID HIV-1 infection increased, the number of technicians participating in the proficiency testing increased over time.

Although corrective actions such as technical support, training and discussions with the laboratory manager were performed, the number of errors for Laboratory D, which accounted for most of the errors during the nine panels, did not decrease. For Laboratory C, most technicians had at least one error over the nine panels. During the retraining and visits, simplification of the procedures was observed, because of the large number of samples being tested. However, for Laboratory A, retraining a technician who was observed making errors helped to overcome some practical issues.

Corrective actions after each EQA panel and retraining of all technicians contributed to a significant reduction of errors over time. Technical assistance decreased in 2013 because of logistical problems faced by the reference laboratory. As a consequence, the number of errors during this period increased, showing the importance of technical assistance to ensure quality testing.

These results reinforce observations made by other studies that training and experience significantly affects the accuracy of proficiency panel testing over time.^22,23,24^ Furthermore, benefits such as relationship strengthening and communication improvement within the laboratories was observed after the implementation of this EQA programme, as observed in other studies.^25^

One of the limitations of our study is that we were not able to follow up with the technicians during all the panels and new technicians were enrolled later in the programme, which might have contributed to the increased number of errors. No sociodemographic data, such as gender, education or how long the technician had been familiar with the procedure and number of panels tested were collected. Thus, our study could not assess the association of such factors with errors observed over the time that the programme was evaluated.

A continuous national quality assessment control programme that includes international panels is important for control of the analytical aspects of EID testing for HIV. This EQA programme ensures quality testing at both the technician and laboratory levels. As indicated by this evaluation, improvement in the quality of testing for EID of HIV for the laboratories from 2011 to 2014 was observed. Our results show that both the National EQA Program for EID of HIV testing model of evaluating technicians, as well as the laboratory, are successful in ensuring the quality of results.
